# Genetic enhancement of palmitic acid accumulation in cotton seed oil through RNAi down‐regulation of *ghKAS2* encoding β‐ketoacyl‐ACP synthase II (KASII)

**DOI:** 10.1111/pbi.12598

**Published:** 2016-09-07

**Authors:** Qing Liu, Man Wu, Baolong Zhang, Pushkar Shrestha, James Petrie, Allan G. Green, Surinder P. Singh

**Affiliations:** ^1^ CSIRO Agriculture & FoodCanberraACTAustralia; ^2^ State Key Laboratory of Cotton BiologyCotton Research InstituteChinese Academy of Agricultural SciencesAnyangChina; ^3^ Jiangsu Provincial Key Laboratory of AgrobiologyJiangsu Academy of Agricultural SciencesNanjingChina

**Keywords:** palmitic acid, KASII, cotton seed oil, RNAi, fatty acids, TAG

## Abstract

Palmitic acid (C16:0) already makes up approximately 25% of the total fatty acids in the conventional cotton seed oil. However, further enhancements in palmitic acid content at the expense of the predominant unsaturated fatty acids would provide increased oxidative stability of cotton seed oil and also impart the high melting point required for making margarine, shortening and confectionary products free of *trans* fatty acids. Seed‐specific RNAi‐mediated down‐regulation of β‐ketoacyl‐ACP synthase II (KASII) catalysing the elongation of palmitoyl‐ACP to stearoyl‐ACP has succeeded in dramatically increasing the C16 fatty acid content of cotton seed oil to well beyond its natural limits, reaching up to 65% of total fatty acids. The elevated C16 levels were comprised of predominantly palmitic acid (C16:0, 51%) and to a lesser extent palmitoleic acid (C16:1, 11%) and hexadecadienoic acid (C16:2, 3%), and were stably inherited. Despite of the dramatic alteration of fatty acid composition and a slight yet significant reduction in oil content in these high‐palmitic (HP) lines, seed germination remained unaffected. Regiochemical analysis of triacylglycerols (TAG) showed that the increased levels of palmitic acid mainly occurred at the outer positions, while C16:1 and C16:2 were predominantly found in the sn‐2 position in both TAG and phosphatidylcholine. Crossing the HP line with previously created high‐oleic (HO) and high‐stearic (HS) genotypes demonstrated that HP and HO traits could be achieved simultaneously; however, elevation of stearic acid was hindered in the presence of high level of palmitic acid.

## Introduction

Cottonseed oil is a highly polyunsaturated vegetable oil because more than half of its total fatty acid content is linoleic acid (C18:2^Δ9,12^) that is oxidatively unstable and makes it unsuitable for direct food applications (Jones and King, 1993; O'Brien, 2002). Partial hydrogenation that converts much of linoleic acid into monounsaturated and saturated fatty acids has often been used for whole‐food applications, such as in providing hard stocks with high melting point for margarine and shortening production. However, *trans* fatty acids are commonly produced as a by‐product of the partial hydrogenation process and have been increasingly recognised to have significant cholesterol‐raising properties and increase the risk of cardiovascular disease based on evidence derived from epidemiologic and clinical studies (Mozaffarian *et al*., 2006; Oomen *et al*., 2001; de Souza *et al*., 2015). A recent systematic review and meta‐analysis of observational studies in the past 10 years, including 41 studies of the association between saturated fat intake and health outcomes, covering more than 300 000 people, and 20 studies of *trans* fat intake and health outcomes that covered more than 200 000 people, have found positive associations between consumption of *trans* fat and total coronary heart disease (CHD) and fatal CHD, but not between consumption of saturated fat and CHD, cardiovascular disease (CVD), stroke and type 2 diabetes (de Souza *et al*., 2015). Although further research on saturated fatty acids is required, there is no doubt that the decades trading saturated fats for *trans* fat have had an enormous influence on the rising incidences of heart disease and many of the so‐called metabolic syndromes. Alternative cotton seed oils with similar functionality to partially hydrogenated oil could therefore be nutritionally desirable.

Cotton seed oil rich in oxidatively stable fatty acids, such as oleic acid (C18:1^Δ9^), stearic acid (C18:0) and palmitic acid (C16:0), would meet this purpose. Palmitic acid makes up approximately 25% of the total fatty acids in the conventional cotton seed oil, and its further enhancement is anticipated to not only increase the oxidative stability of cotton seed oil by offsetting the instability of linoleic acid but also impart the high melting point required for making such products as margarine, shortening and confectionary products free of *trans* fatty acids (Neff and List, 1999; Nzikou *et al*., 2009).

Tropical oils such as palm oil contain about 50% of palmitic acid, which is increasingly being used as an alternative to partially hydrogenated vegetable oils in baking and processed food applications (Hayes and Pronczuk, 2010; L'Abbe *et al*., 2009). The mid‐fraction of a palm oil (PMF) rich in symmetrical palmitate triglycerides (POP) has also been most commonly used to formulate cocoa butter substitute by interesterification with oils derived from stearate‐rich tropical oils (L'Abbe *et al*., 2009; Zaliha *et al*., 2004). Oil palm plantings have continued to expand in recent years, in response to rapidly increasing demand for food oil, as well as for biofuel. As the conversion of virgin agricultural land to palm plantation in tropical countries has become an ever more sensitive issue, development of cotton seed oil with similar fatty acid composition and functionality to palm oil may offer an attractive alternative because of its continuous availability that is driven by the continuing demand for cotton fibre.

In higher plants, the plastid is the major site of *de novo* fatty acid biosynthesis pathway, generating fatty acyl precursors for fatty acids of different chain lengths and saturation levels. The *de novo* fatty acid biosynthesis is performed by a complex of soluble proteins known as fatty acid synthases (FAS), with the β‐ketoacyl‐ACP synthase (KAS) enzyme family catalysing the elongation of malonyl‐acyl carrier protein (malonyl‐ACP) by reiteratively adding C2 units to a growing fatty acyl chain through Claisen condensation (Ohlrogge and Jaworski, 1997). KASIII catalyses the condensation of C2‐CoA to C4, while KASI prefers C4‐ to C14‐ACP substrates leading to the production of palmitoyl‐ACP that is then elongated to stearoyl‐ACP by KASII and subsequently desaturated to form oleoyl‐ACP by Δ9 stearoyl‐ACP desaturase (SAD). The saturated fatty acids, mostly palmitic acid in cotton seed, can be cleaved from palmitoyl‐ACP by the action of the palmitoyl‐ACP thioesterase (FatB), allowing for transportation of free palmitic acid into cytoplasm where it becomes available for further desaturation and triacylglycerol (TAG) assembly.

Because palmitoyl‐ACP is the substrate for two major activities, KASII and FatB, it represents a key branch point in fatty acid biosynthesis (Cahoon and Shanklin, 2000). Most fatty acids in the seed oil pass through a C16 form during biosynthesis, and the content of palmitic acid remaining in the final cotton seed oil is therefore determined, to a large extent, by the competing action of FatB and KASII (Cahoon and Shanklin, 2000; Martz *et al*., 2006). These enzymes therefore represent possible targets for genetic manipulation of palmitic acid levels in cotton seed oil (Aslan *et al*., 2015; Pidkowich *et al*., 2007; Sun *et al*., 2014).

Genetic engineering of *FatB* has previously been successful in raising or lowering palmitic acid levels in seeds of other plants. Overexpression of the Arabidopsis *FatB1* in seeds resulted in a nearly four fold increase in seed palmitic acid content (Dormann *et al*., 2000). Transgenic expression of a *FatB* gene derived from *Cuphea hookerinana* resulted in raised palmitic acid from 6% to 34% in rapeseed oil (Dehesh *et al*., 1996). An even more effective approach for raising palmitic acid was demonstrated by RNAi down‐regulation of *KAS2* that encodes KASII in Arabidopsis where palmitic acid level was raised to as high as 53% beyond which level the seeds were aborted (Pidkowich *et al*., 2007). It is assumed that the significantly lowered KASII activity resulting from the attenuated *KAS2* transcription reduces the flow of metabolites from palmitoyl‐ACP to stearoyl‐ACP, causing a significant accumulation of the palmitoyl‐ACP and consequently enriched palmitic acid content in TAG.

We have employed a similar approach to raise palmitic acid in cotton seed oil. In this report, we describe the characterisation of two different *KAS2* genes from developing cotton embryos and their RNAi down‐regulation that led to substantial enhancement of palmitic acid accumulation. Attempts have also been made to combine the high‐palmitic (HP) trait by crossing with either or both high‐oleic (HO) and high‐stearic (HS) traits that have been previously generated using RNAi‐mediated gene down‐regulation of *ghFAD2‐1* or *ghSAD‐1*, respectively (Liu *et al*., 2002).

## Results

### Isolation of two different *KAS2* cDNAs from developing cotton seed

**Figure 1:**
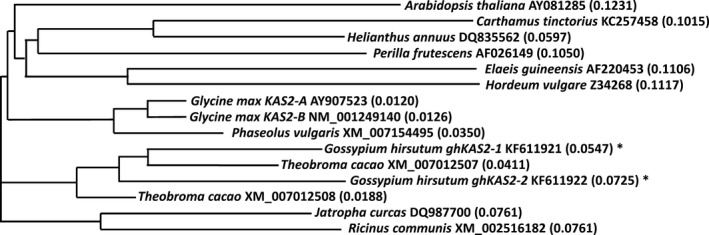
Phylogenetic comparison of cotton *ghKAS2* gene family and orthologous *KAS2s* from some selected plant species. The phylogenetic tree was constructed from multiple sequence alignment of KASII protein from the GenBank generated by the AlignX module of Vector NTI suite using neighbour‐joining clustering algorithm in Clustal X2 program. The GenBank accession numbers and the sources of *KAS2* genes and bootstrap values of the clusters are indicated in phylogram. The sequences of *ghKASII* from this study are marked with an asterisk.

From a cDNA library derived from developing embryos of upland cotton (*Gossypium hirsutum* L.) cv DP‐16, two distinct cDNAs were isolated and designated as *ghKAS2‐1* (GenBank accession No. KF611921) and *ghKAS2‐2* (GenBank accession No. KF611922). Full length sequences of these cDNAs were completed by 5′ race using RNAs derived from developing embryos as templates. The complete *ghKAS2‐1* is 2268 bp long, encoding 577 amino acids. The *ghKAS2‐2* cDNA is 2192 bp long, encoding 526 amino acids. The putative polypeptides encoded by the coding region of *ghKAS2‐1* and *ghKAS2‐2* showed 78.7% identity to each other. The phylogenetic relationship of these two genes and their orthologs in a selected number of plant species is illustrated in Fig. 1. It appears that *ghKAS2‐1* and *ghKAS2‐2* clustered together with their ortholog derived from *Theobroma cacao* that is taxonomically closely related to cotton. By searching the *G. raimondii* genome database, *ghKAS2‐1* was mapped onto chromosome 13 and its coding region was interrupted by 12 introns and the 13th intron was found in the 3′ UTR. *ghKAS2‐2* was mapped onto chromosome 4, and its coding region was interrupted by 12 introns. The position and length of introns were relatively conserved between *ghKAS2‐1* and *ghKAS2‐2*. It is noteworthy that about half of the *ghKAS2‐1* cDNA clones isolated from the cotton developing embryo cDNA library contained intron 12 that was not properly spliced. It is not known whether this constitutes one level of post‐transcriptional regulation and is responsible for the higher level of palmitic acid in cotton seed relative to many other temperate oilseed crops.

### RNAi construct designing and selection of primary transgenic cotton plants

As detailed in the Experimental procedures, a chimeric DNA sequence consisting of an inverted repeat structure of *ghKAS2‐1* was used as the trigger DNA sequence in an RNAi cassette. Instead of a typical intron sequence, the upstream adjacent sequence of the 400‐bp sense arm of the inverted repeat was used as a spacer separating the head to head inverted repeat. Although the trigger sequence was designed based on the *ghKAS2‐1*, it was chosen as the most conserved region of the gene. The trigger sequence has 87.5% homology with *ghKAS2‐2,* with several stretches of continuous homologous regions longer than 21 bp, enabling it to achieve a level of cross‐silencing of *ghKAS2‐2*. A seed‐specific promoter derived from soya bean lectin gene *LEC1* (Cho *et al*., 1995) was used to drive the transgene. Six independent cotton lines transformed with the *RNAi‐ghKAS2‐1* construct were regenerated and allowed to grow to maturity. Four of the six lines were male sterile, a much higher frequency than seen in other typical cotton transformations in our laboratory, suggesting a possible impact of severe *KAS2* down‐regulation rather than a side effect of tissue culture. The other two fertile lines, KIR‐1 and KIR‐10, were carried through for further molecular and biochemical analyses. PCR was first performed to confirm the transgene presence, followed by Southern blot analysis with multiple restriction enzymes on the T_1_ primary transgenic plants using the promoter region of the transgene as a probe. Single restriction bands were observed in both KIR‐1 and KIR‐10, indicating single transgene insertions (data not shown). Under glasshouse conditions, neither of these two lines exhibited any growth abnormalities nor was there any apparent penalty on plant vigour or seed yield.

**Figure 2:**
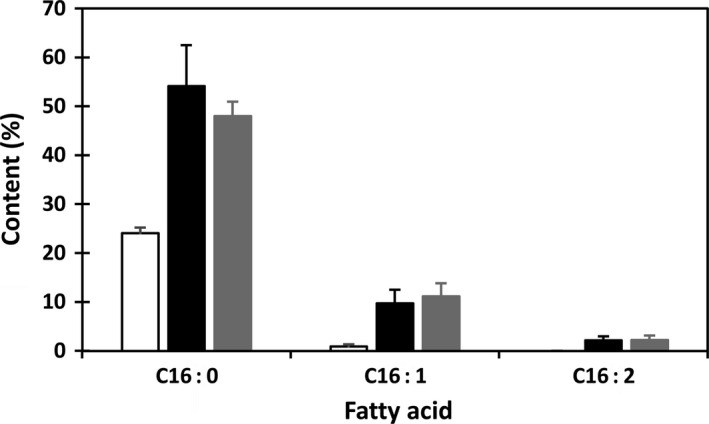
Accumulation of C16 fatty acids in the primary transgenic plants of transgenic lines KIR‐1 and KIR‐10. WT (open box); KIR‐1 (closed box) and KIR‐10 (shaded box).

Analysis of fatty acid composition was carried out along with the selection and establishment of homozygous transgenic plants. In comparison with wild type (WT) and null segregants, fatty acid profiles of the transgenic seeds derived from KIR‐1 and KIR‐10 have altered significantly, featuring enhanced C16 fatty acids with concomitant reduction in C18 fatty acids. Fig. 2 shows the content of palmitic acid and C16 unsaturated fatty acids in the cotyledons of 65 and 51 randomly selected individual T_2_ seeds in KIR‐1 and KIR‐10, respectively. Among the T_2_ seeds, the segregation of null seeds is evident as they have similar fatty acid composition to WT plants, while all other seeds showed raised palmitic acid at various levels, with the majority exceeding 50% of total fatty acids in both transgenic lines. The ratios of transgenic seeds to null segregants for KIR‐1 (54 : 11) and KIR‐10 (49 : 9) fitted a 3 : 1 ratio expected for the segregation of a single dominant gene (*x* ^2^ = 0.19 and 0.10, respectively), consistent with the molecular assessment of single copy transgenes as confirmed by Southern blot analysis. In KIR‐1, a single seed with the highest accumulation of total C16 fatty acids reached 85% of total fatty acids. Palmitoleic acid was raised to as high as 11% of total fatty acids. C16:2 that is normally undetectable in cotton seed accounted for up to 3.3% of total fatty acids. Compared to KIR‐1, the extent of increase in palmitic acid and unsaturated C16 fatty acid levels in KIR‐10 seeds is relatively consistent among the transgenic T_2_ seeds. Most of the transgenic seeds showing palmitic acid level between 45% and 50%. Similar to KIR‐1, the sum of palmitoleic acid and C16:2 was also increased to about 15% of total fatty acids in individual KIR‐10 seeds. As the result, the contents of C18 fatty acids were all reduced, except for *cis*‐vaccenic acid (C18:1^Δ11^) that is the elongation product of palmitoleic acid. There is an approximate doubling of *cis*‐vaccenic acid content in both KIR‐1 and KIR‐10 lines compared to untransformed control.

The T_2_ seeds with significantly raised palmitic acid were selected and germinated for plant establishment and assessment of the inheritance stability of the gained HP trait in the progenies of both KIR‐1 and KIR‐10. Fifteen individual mature T_3_ seeds borne on each of the established T_2_ plants were subjected to analysis of fatty acid composition. There were clear segregation of null seeds with WT‐like fatty acid profile among the T_3_ seeds, and the homozygous HP lines were able to be established at T_4_ generation for both KIR‐1 and KIR‐10.

The fatty acid profiles of T_2_ (null excluded), T_3_ and T_4_ seeds were tabulated in Table 1. In all the three generations, the increased contents of palmitic acid and its derivative C16 unsaturated fatty acids were clearly a prominent feature and consistent among generations. The increase of C16 fatty acids was at the expense of all three major C18 fatty acids, including stearic, oleic and linoleic acids. In KIR‐1, distinct from the high variability in primary transgenic seeds, the palmitic acid accumulation in T_3_–T_4_ generations was more consistent among individual seeds without significant variation.

**Table 1:** Fatty acid composition of transgenic cotton expressing RNAi‐ghKAS2‐1 in three successive progeny generations

Generation	Genotype	C14:0	C16:0	C16:1	C16:2	C18:0	C18:1^Δ9^	18:1^Δ11^	C18:2
T_2_	Null	0.2 ± 0.1	24.1 ± 1.1	0.9 ± 0.4	0.0 ± 0.0	2.1 ± 0.6	13.3 ± 2.9	0.8 ± 0.6	58.1 ± 3.1
	KIR‐1	0.2 ± 0.1	54.1 ± 8.4	9.7 ± 2.7	2.2 ± 0.8	1.2 ± 0.5	3.5 ± 2.0	1.5 ± 0.4	26.8 ± 7.2
	KIR‐10	0.2 ± 0.1	48.0 ± 2.9	11.2 ± 2.7	2.2 ± 0.9	1.0 ± 0.1	3.9 ± 1.4	1.7 ± 0.2	31.3 ± 4.8
T_3_	KIR‐1	0.2 ± 0.1	51.0 ± 2.0	11.2 ± 2.1	2.8 ± 0.5	1.0 ± 0.0	2.3 ± 0.1	1.5 ± 0.0	29.2 ± 3.4
	KIR‐10	0.3 ± 0.1	49.5 ± 1.2	10.7 ± 2.3	2.1 ± 0.7	1.0 ± 0.1	3.7 ± 1.3	1.6 ± 0.1	30.4 ± 2.1
T_4_	KIR‐1	0.2 ± 0.1	50.2 ± 1.7	10.9 ± 2.0	2.2 ± 0.7	1.1 ± 0.1	4.7 ± 1.8	1.8 ± 0.3	28.4 ± 2.8
	KIR‐10	0.2 ± 0.1	48.9 ± 1.8	11.8 ± 2.2	2.61 ± 0.7	1.1 ± 0.1	4.0 ± 1.6	1.7 ± 0.2	29.0 ± 2.5

Mean ± Std (*n* = 3).

### Oil content analysis and seed germination test

Assessment of oil content was carried out in mature homozygous seeds harvested from the T_4_ and T_5_ HP lines and WT plants that were grown alongside each other under controlled greenhouse condition. Across the two generations, there was a slight yet significant decline of oil content in both HP lines (*P* < 0.05), while there is no significant variation between KIR‐1 and KIR‐10 (Table 2). Two‐way analysis of variance (ANOVA) revealed significant variation in oil content neither between generations nor interaction between genotype and generation (Table S1).

**Table 2:** Oil content analysis of T_4_ and T_5_ cotton seeds and the germination rate of T_4_ cotton seeds at two different temperatures (18 °C and 28 °C)

Genotype	Oil content (%)		Germination rate (%)	
	T_4_	T_5_	18 °C	28 °C
Coker 315	22.8 ± 0.7_a_	23.6 ± 0.5_a_	81.7 ± 4.3_a_	97.5 ± 3.2_b_
KIR‐1	21.3 ± 1.1_b_	22.0 ± 0.1_b_	80.0 ± 4.7_a_	96.7 ± 2.7_b_
KIR‐10	21.3 ± 0.4_b_	21.5 ± 0.4_b_	83.3 ± 6.1_a_	95.8 ± 3.2_b_

Mean ± Std (oil content analysis, *n* = 3; germination test, *n* = 4). Columns with different letters represent significant differences (LSD) at p < 0.05.

Germination response to temperature of homozygous T_4_ seeds of KIR‐1 and KIR‐10 lines was evaluated at cool (18 °C) and warm (28 °C) temperature regimes. All the seeds derived from both WT and HP lines showed significantly higher germination rate at 28 °C compared to 18 °C, but there was no significant variation among cotton lines (*P* < 0.05; Table 2; Table S2).

### Real‐time RT‐PCR gene expression

**Figure 3:**
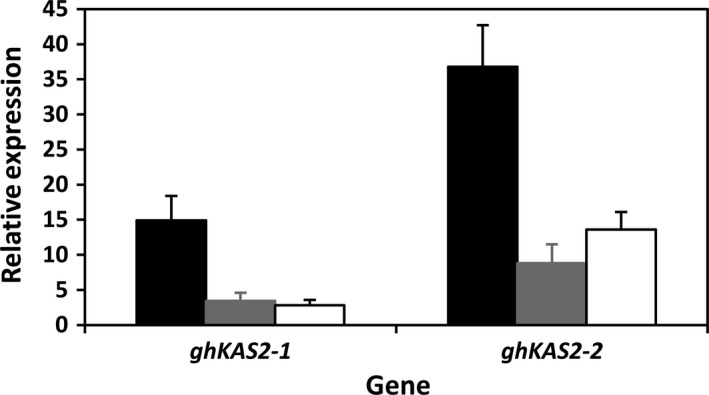
Real‐time RT‐qPCR analysis of *ghKAS2‐1* and *ghKAS2‐2* in immature cotton embryos at mid‐developmental stage (30 DAA). Coker315 (closed box); KIR‐1 (shaded box) and KIR‐10 (open box).

To confirm the molecular basis of the alteration in fatty acid composition in KIR‐1 and KIR‐10, real‐time quantitative PCR (RT‐qPCR) was performed to evaluate the transcription level of the target *KAS2* gene using template RNAs derived from mid‐maturity developing embryos as previous studies have established that this is the most active period of fatty acid biosynthesis and oil accumulation (Liu *et al*., 1999). As shown in Fig. 3, the reduction in the expression of both *ghKAS2‐1* and *ghKAS2‐2* genes in the T_4_ homozygous transgenic lines was evident compared to WT control. It is also noteworthy that expression of *ghKAS2‐1* appeared to be lower than *ghKAS2‐2* in developing cotton seeds. This result is consistent with our observation that cDNA library screening yields 3 times more clones of *ghKAS2‐2* than *ghKAS2‐1*. Taken together, we may assume that these two genes do not have equal contributions to the conversion of palmitic acid to stearic acid. Although the *ghKAS2‐1* sequence alone was used as the RNAi trigger sequence, the expression of both *ghKAS2‐1* and *ghKAS2‐2* has been effectively attenuated.

### Lipid analysis of HP cotton seed oil

**Figure 4:**
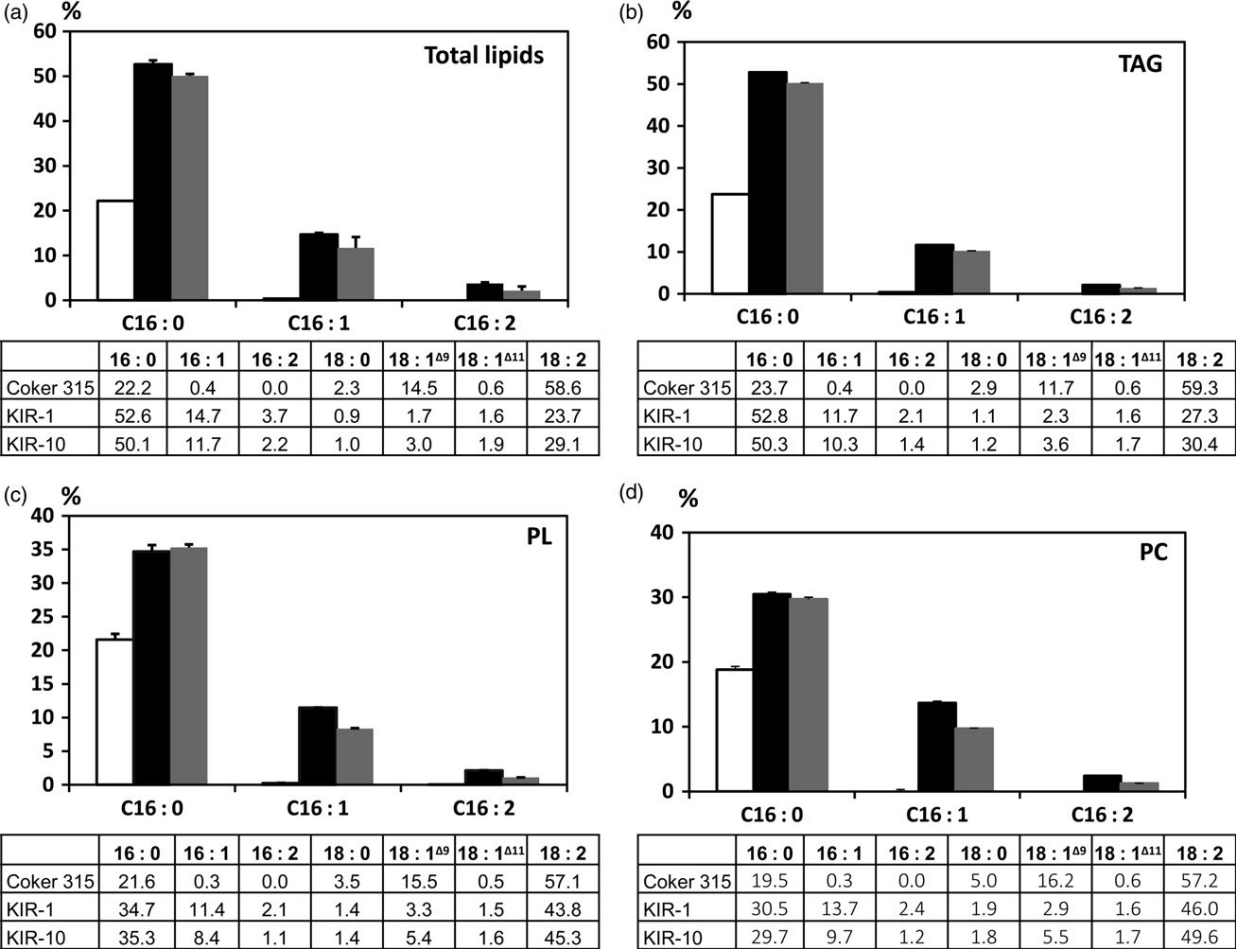
Fatty acid composition (%) of total lipids (a), TAG (b), PL (c) and PC (d). HP cotton seed oil derived from KIR‐1 (closed box) and KIR‐10 (shaded box) were compared to WT (Open box). Lipids were extracted from homozygous T_4_ seeds and fractionated by TLC to generate neutral and polar lipid fractions. Data are presented as mean ± SD (*n* = 3). The full fatty acid composition of cotton seed oil is tabulated below the bar diagram.

Palmitic acid content in the total lipids extracted from mature whole cotton seeds of KIR‐1 and KIR‐10 was measured as 52.6% and 50.1%, respectively, compared to 22.2% in WT (Fig. 4). In the TAG fraction, palmitic acid content in KIR‐1 and KIR‐10 seeds was 52.8% and 50.3% of total fatty acids, respectively. Similarly, palmitic acid content in the polar lipid (PL) fraction of KIR‐1 and KIR‐10 seed oil was 34.7% and 35.3%, respectively, compared to 21.6% in WT; palmitic acid content in the phosphatidylcholine (PC) fraction of KIR‐1 and KIR‐10 was 30.5% and 29.7%, respectively, in contrast to 19.5% in WT (Fig. 4). The increase in unsaturated C16 fatty acids was consistent among TAG, total PL and PC. Linoleic acid decreased with a concomitant increase in palmitic acid and other C16 fatty acids, more evident in TAG compared to total PL and PC. The relatively smaller alteration of fatty acid composition in total PL and PC might explain why there was little impact on seed germination and plant growth by RNAi down‐regulation of *KAS2* expression in cotton seed.

### Positional analysis of palmitic acid

**Figure 5:**
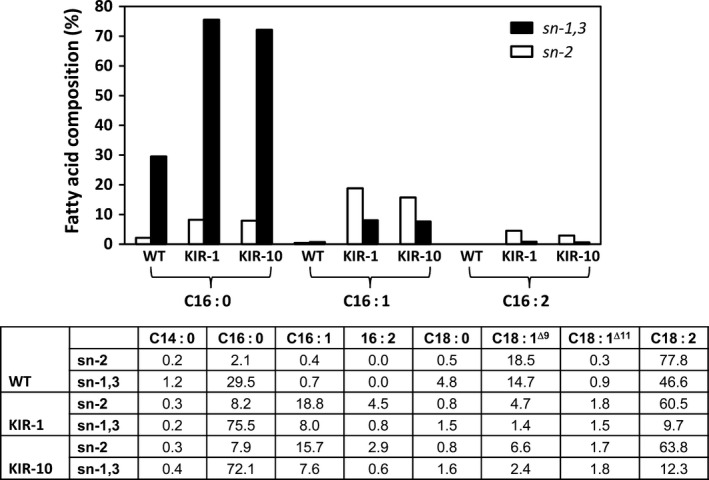
*sn* Positional distribution of fatty acids in TAG fraction of cotton seed oil derived from HP cotton lines, KIR‐1 and KIR10, compared to the untransformed control Coker 315 (WT). *sn*‐1,3 (closed box); *sn*‐2 (open box). The full fatty acid composition of the specified *sn* position is tabulated below the bar diagram.

To determine the regiospecificity of palmitic acid and its two C16 derivative fatty acids, palmitoleic acid and C16:2, in KIR‐1 and KIR‐10, the TAG fraction was isolated from the homozygous T_4_ seeds and digested with appropriate lipase that preferentially cleaves fatty acid from the outer positions (*sn*‐1 and *sn*‐3), releasing two free fatty acids and a 2‐monoacylglycerol (2‐MAG) molecule retaining the *sn*‐2 acyl chain. In KIR‐1 and KIR‐10, consistent with WT, the outer positions of TAG were predominantly occupied by palmitic acid, while the *sn*‐2 position was mainly occupied by unsaturated fatty acids, mostly linoleic acid. Palmitoleic acid and C16:2 were found in both *sn*‐2 and outer positions, with some degree of preference for *sn*‐2 (Fig. 5). This was consistent with the general observation that, in plant seed oil, saturated fatty acids preferentially occupy the outer positions in TAG, whereas unsaturated fatty acids are predominantly found in *sn*‐2 position.

**Figure 6:**
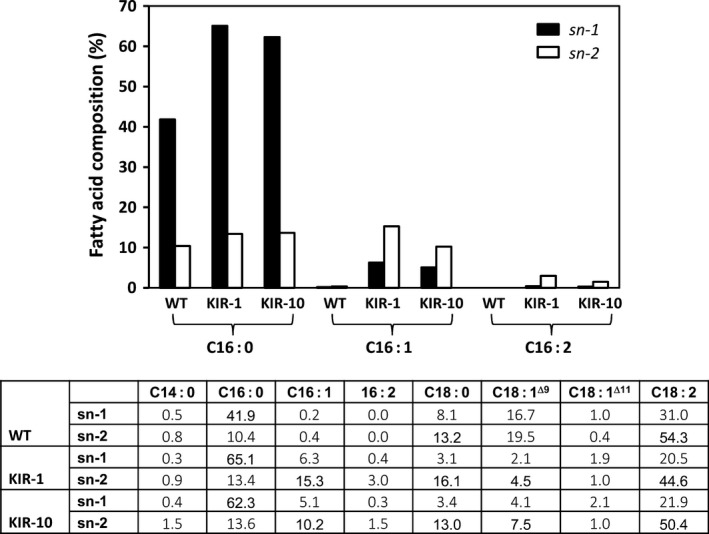
*sn* Positional distribution of fatty acids in PC fraction of cotton seed oil derived from HP cotton lines, KIR‐1 and KIR10, compared to the untransformed control Coker 315 (WT). *sn‐*1 (closed box); *sn‐2* (open box). The full fatty acid composition of the specified *sn* position is tabulated below the bar diagram.

For both HP lines, we also analysed the positional distribution of fatty acids on PC, an important intermediate in TAG synthesis and membrane constituents. The isolated PC fraction was digested with phospholipase A2, an enzyme that preferentially cleaves fatty acids from the *sn*‐2 of PC, releasing a free fatty acid and a lyso‐PC molecule retaining the *sn*‐1 acyl chain. This positional analysis showed that consistent with WT, the *sn*‐1 position was predominantly occupied by palmitic acid, while *sn*‐2 position was mainly occupied by unsaturated fatty acids (Fig. 6). Palmitoleic acid and C16:2 were found on both *sn*‐1 and *sn*‐2 positions, with a clear preference for *sn*‐2. It was also clear that palmitic acid in KIR‐1 and KIR‐10 was mainly found on *sn*‐1 position, while its increase on *sn*‐2 position was relatively minor.

### Incorporation of HP trait into HS, HO and HSO cotton genotypes

**Figure 7:**
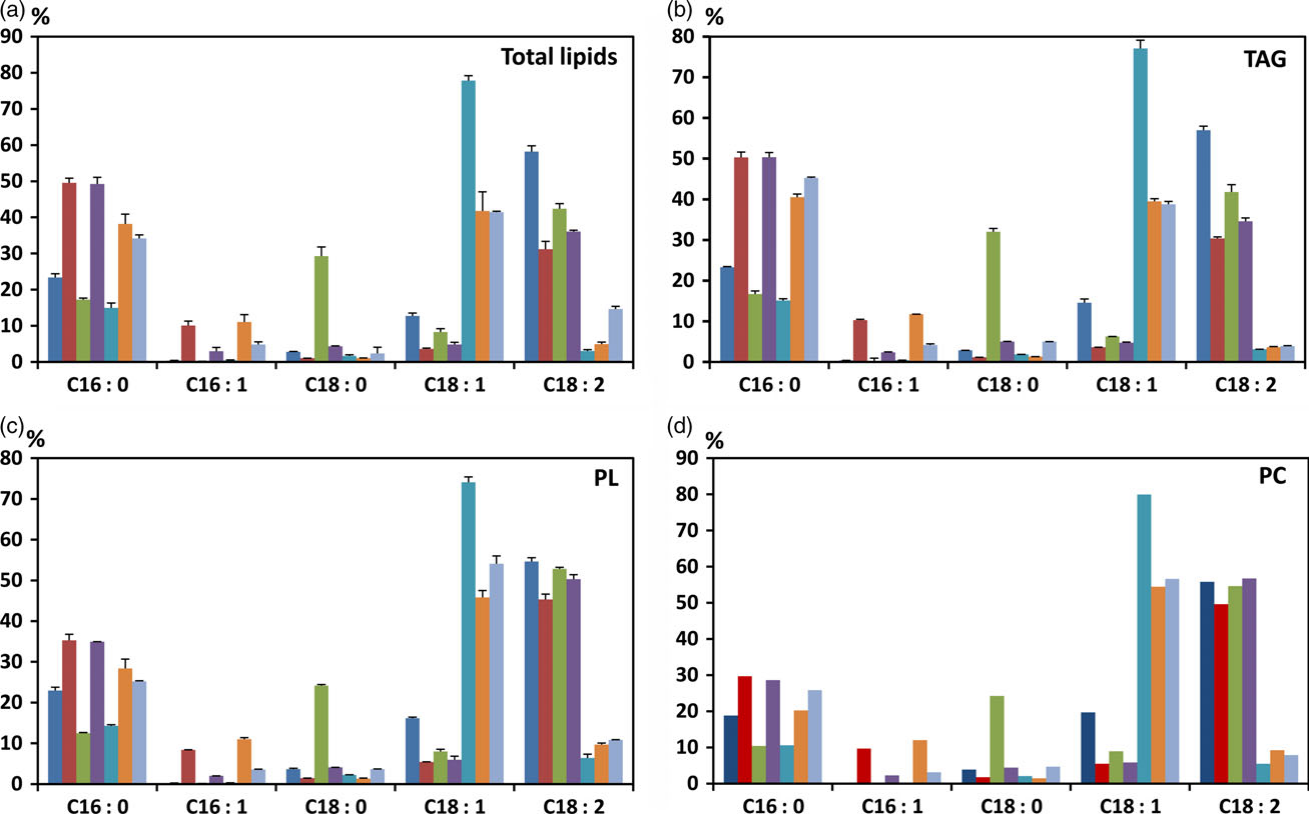
Fatty acid composition of total lipids (a), TAG (b), PL (c) and PC (d) in various cotton seed oil featuring Coker315 (blue), HP (red), HS (green), HP × HS (purple), HO (turquoise), HP × HO (orange), HP × (HS × HO) (grey) genotypes. Total lipids were fractionated by TLC to generate neutral and polar lipids. Data are presented as mean ± SD (*n* = 3).

To incorporate the HP trait into some other genotypes with genetically altered fatty acid profiles, KIR‐10 was crossed with previously generated HO, HS lines and their homozygous hybrid (HSO) (Liu *et al*., 2002). The fatty acid profiles of the total lipids, TAG, PL and PC fractions in the F_1_ hybrid seeds derived from a cross between KIR‐10 and a HS line, HS‐35, are shown in Fig. 7. Palmitic acid in the F_1_ seeds was increased to a similar level as in its HP parent in the total lipids and TAG fraction, but its increase in PL and PC fractions was not as prominent. Correspondingly, the reduction in linoleic acid was also significantly more prominent in total lipids and TAG fraction than in polar lipids. In contrast to the high level accumulation of palmitic acid, the stearic acid level in all the four lipid pools in the HP x HS F_1_ seeds remains low, about 4%, which is significantly higher than WT, but much lower than its HS‐35 parent.

The F_1_ hybrid seeds derived from a cross between KIR‐10 with a HO cotton line, HO‐30, showed the simultaneous increase of both palmitic and oleic acids, mainly at the expense of linoleic acid (Fig. 7). The average level of palmitic acid in the total seed lipids of the HP × HO cross was 41.7%, compared to 50% in the HP parent. The level of oleic acid in the HP × HO cross averaged 38.2% of total seed fatty acids, which in absolute terms is only half of the 78% oleic in the HO parent. However, in the context of the reduced pool of C18 fatty acids in the presence of the HP trait, the 38.2% of oleic acid does represent approx. 85% of the total C18 fatty acids, which is approaching the very high preponderance of oleic acid in the C18 fatty acid pool of the HO parent (94%). Reflecting the effect of *FAD2* silencing, the linoleic acid content in the HP × HO hybrid appeared to be at a similar level to that in its HO parent, maintaining below 5%, while its HP parent contained 31.2% linoleic acid. Such a profile was consistent in the TAG, total PL and PC fractions. It is also worthy of noting that the ratio of palmitic/oleic acids was substantially lower in total PL and PC fraction, compared to total lipids and TAG fraction. Palmitoleic acid level remained at a similar level among the four lipid pools examined.

The hybrid (HPSO) was obtained by crossing KIR‐10 with a homozygous HO/HS cotton plant (HO/HS‐9) that was generated through crossing HO‐30 and HS‐35. In its total lipids, similar to what have been observed in the HP × HO and HP × HS, the combination of HP and HO traits was evident, but the stearic acid level was only moderately increased, averaging 3.4%, marginally, yet significantly higher than WT (2.8%). Linoleic acid remained low, about 5%, a similar level to the HO and HP × HO genotypes. Such a trend was consistent in the other three lipid pools, including TAG, PL and PC fractions. This hybrid also showed the common feature with HP and HPO genotypes that in PL and PC the ratio of palmitic acid *vs* linoleic acid was substantially lower than that in total lipids and TAG fraction.

### Regiospecific distributions of fatty acids in the hybrids combining HP and other genotypes with altered fatty acids

The regiospecific distribution of fatty acids on TAG and PC molecules in the hybrids incorporating the HP trait into HS, HO and HSO lines was also analysed and shown in Tables 3 and 4, respectively. In the TAG of high saturate lines, that is HP, HS and HPS, the increases in palmitic acid and stearic acid were mainly concentrated on the *sn*‐1 and *sn*‐3 position (Table 3). This was consistent with PC except that stearic acid seemed to prefer *sn*‐2 in the WT, HP and HO lines where it was present at low levels (Table 4). A general preference of *sn*‐2 position by unsaturated fatty acids, including palmitoleic acid, C16:2, oleic acid and linoleic acid, was evident in both TAG (Table 3) and PC. However, oleic acid was found on all three *sn* positions when its accumulation reached very high level in HO‐30.

**Table 3:** Positional distribution of fatty acids on TAG in different cotton genotypes containing altered fatty acid composition

Sample	Position	C14:0	C16:0	C16:1	16:2	C18:0	C18:1^Δ9^	C18:1^Δ11^	C18:2
Coker 315	*sn*‐2	0.2	2.5	0.4	0.0	0.7	16.9	0.3	78.8
	*sn*‐1 + 3	1.0	36.1	0.5	0.1	5.3	11.7	0.8	42.2
KIR‐10	*sn*‐2	0.3	7.9	15.7	2.9	0.8	6.6	1.7	63.8
	*sn*‐1 + 3	0.4	72.1	7.6	0.6	1.6	2.4	1.8	12.3
HS‐35	*sn*‐2	0.5	4.9	0.3	0.0	5.5	12.9	0.0	75.3
	*sn*‐1 + 3	0.5	26.3	0.4	0.1	41.6	6.4	0.3	21.3
HPS	*sn*‐2	0.2	6.5	3.9	0.5	0.8	8.7	0.6	78.5
	*sn*‐1 + 3	0.7	72.8	2.4	0.2	7.8	3.9	0.6	9.0
HO‐30	*sn*‐2	0.1	1.2	0.4	0.0	0.5	92.6	0.6	4.5
	*sn*‐1 + 3	1.7	19.1	1.8	0.0	4.1	65.2	1.1	5.3
HPO	*sn*‐2	0.1	2.6	17.2	0.0	0.4	69.5	2.2	7.9
	*sn*‐1 + 3	0.3	58.4	8.9	0.0	2.2	24.8	1.8	1.8
HPSO	*sn*‐2	0.1	3.3	6.0	0.0	0.6	80.1	1.0	8.7
	*sn*‐1 + 3	0.5	64.6	3.5	0.0	7.5	18.9	0.5	1.4

HPS = KIR‐10 × HS‐35; HPO = KIR‐10 × HO‐30; HPSO = KIR‐10 × (HS‐35 × HO‐30).

**Table 4:** Positional distribution of fatty acids on PC in different cotton genotypes containing altered fatty acid composition

Sample	Position	C14:0	C16:0	C16:1	C16:2	C18:0	C18:1^Δ9^	C18:1^Δ11^	C18:2
Coker 315	*sn*‐1	0.7	48.1	0.1	0.0	10.7	13.1	1.1	25.6
	*sn*‐2	2.0	18.6	0.4	0.0	21.0	14.5	0.3	41.7
KIR‐10	*sn*‐1	0.4	62.3	5.1	0.3	3.4	4.1	2.1	21.9
	*sn*‐2	1.5	13.6	10.2	1.5	13.0	7.5	1.0	50.4
HS‐35	*sn*‐1	0.3	21.2	0.1	0.0	49.0	5.8	0.2	22.1
	*sn*‐2	0.5	7.0	0.3	0.0	11.5	11.5	0.1	67.9
HPS	*sn*‐1	0.3	61.2	1.0	0.0	9.4	3.7	0.7	22.8
	*sn*‐2	0.7	11.0	2.7	0.3	12.6	7.6	0.3	63.3
HO‐30	*sn*‐1	0.5	23.4	0.4	0.0	4.8	65.2	1.4	3.9
	*sn*‐2	0.9	9.8	0.3	0.0	15.5	68.1	0.4	4.4
HPO	*sn*‐1	0.3	43.9	7.0	0.0	2.9	37.1	2.5	5.8
	*sn*‐2	1.1	12.1	10.7	0.2	15.5	50.6	0.7	7.6
HPSO	*sn*‐1	0.4	41.4	5.8	0.0	6.7	39.2	2.2	4.3
	*sn*‐2	1.1	10.2	11.5	0.2	16.5	53.8	0.9	5.8

HPS = KIR‐10 × HS‐35; HPO = KIR‐10 × HO‐30; HPSO = KIR‐10 × (HS‐35 × HO‐30).

## Discussion

Palmitic acid is a common saturated fatty acid widely present in our diet system, and reports claiming and disputing a link between ingestion of palmitic acid and heart disease have been well documented (Chowdhury *et al*., 2014; Martinez‐Ortiz *et al*., 2006; Narang *et al*., 2005). Although the overall benefit or risk of HP oils has not been established, it is clear that HP vegetable oil is a better choice than *trans* fats (de Souza *et al*., 2015). While emphasis has been placed on reducing saturate fatty acids in the main stream vegetable oils, considerable efforts have also been made in raising palmitic acid in numerous vegetable oils because of its essential functional role in structural food lipids and high oxidative stability in *trans*‐free fats.

In soya bean, major alleles in at least five loci have been reported to cause an increase in palmitic acid content ranging between 4% and 40%, and the palmitic acid content has been elevated to above 40% of total fatty acids in lines combining HP mutant alleles (Stoltzfus *et al*., 2000). Some of these HP genotypes have been found to be associated with defective *KAS2* genes. For instance, J10 has a deletion of entire *gmKASIIA*; M22 has a small deletion in an intron of *GmKASIIB,* which results in mistranslation of approximately 80% of *GmKASIIB* transcripts (Anai *et al*., 2012).

Arabidopsis mutant *fab1‐1,* having approximately double the amount of palmitic acid compared to that of WT plants (James and Dooner, 1990), was identified as a Leu‐337Phe substitution in KASII protein that causes instability due to insufficient space for accommodating the imidazole ring of the mutant Phe‐337 residual (Carlsson *et al*., 2002; Wu *et al*., 1994). Enzyme assay on the extracts from *fab1‐1* mutant line revealed a 40% reduction of KASII activity *in vitro*. Seed‐specific down‐regulation of *atKAS2* driven by *Phaseolin* promoter in Arabidopsis illustrated that up to 53% of palmitic acid could be accumulated in seed oil without obvious negative impact on seed development (Pidkowich *et al*., 2007). In this report, we have demonstrated in cotton that it is possible to raise palmitic acid level substantially and mimic an oil palm‐like profile by genetically altering the expression level of *ghKAS2* gene in an otherwise HO genetic background.

KASII enzymes are highly conserved between different plant species, and there is a consensus regarding their role as the general housekeeping fatty acid synthases responsible for the production of most C18 fatty acids in plastids. The characterisation of a T‐DNA knockout mutant of *KAS2* in Arabidopsis suggested that a basic level of KASII activity is essential for normal seed viability because the homozygote mutant genotype was not able to be recovered (Pidkowich *et al*., 2007).

During cotton transformation process, many of the somatic embryos derived from transgenic calli have been aborted at globular stage, which led to substantially fewer number of transgenic plants compared to a standard cotton transformation experiment in our laboratory. In addition to the two fertile transgenic lines we are reporting here, we have also generated a number of male sterile lines at an unusually high rate. When the male sterile lines were pollinated with WT, among a large number of aborted seeds, the recovered viable seeds contained higher level of palmitic acid than those of KIR‐1 and KIR‐10. But the effort has failed to establish a stable transgenic line from their progenies because of the highly variable palmitic acid content and poor seed viability that is likely attributable to the nature of high copy number of *ghKAS2‐RNAi* transgene. Such a result is consistent with the observation in Arabidopsis that strong suppression *atKAS2* resulted in abortion of about one‐fourth of zygotic embryos before torpedo stage, suggesting lethality at homozygosity (Pidkowich *et al*., 2007).

A slight yet significant reduction in oil content was observed in both KIR‐1 and KIR‐10 seeds. It is well established that the energy requirement for the entire seed germination process including seed imbibition, radical protrusion, elongation and initial seedling development prior to photosynthetic autotrophy is provided by the available nutritive reserves of the cotyledons (Snider *et al*., 2014; Turley and Chapman, 2010). As seed oil represents the most energy‐dense storage compound of the quiescent cotton seed, it has been suggested that higher seed oil content would enhance seed germination rate and seedling vigour by providing more chemical energy (Bartee and Krieg, 1974; Snider *et al*., 2014). However, the reduction in seed oil content in both HP lines did not result in compromised seed germination at both cool and warm temperatures. It is particularly interesting that the dramatic increase of palmitic acid that has relatively high melting point compared to unsaturated fatty acids showed a neutral effect on germination rate, which is in sharp contrast to the severe impairment on germination observed in HS cotton seeds generated by RNAi down‐regulation of SAD (Liu *et al*., 2002). There is no doubt that detailed assessments on a range of seed characteristics need to be carried out, especially in field conditions as early season soil temperatures may strongly influence seed germination and seedling establishment.

Similar to the observation in Arabidopsis, a key feature of the HP cotton lines was a significant increase in levels of the so‐called ω7 fatty acids, including palmitoleic acid, C16:2 and *cis*‐vaccenic acid when compared with WT cotton. Palmitoleate is found only in trace amounts in most plant tissues, whereas oleate is the main product of plastidial fatty acid biosynthesis. The increased production of C16 unsaturated fatty acids may suggest the presence of a broad substrate specificity of SAD that acts on palmitoyl‐ACP to produce palmitoleic acid that could be either further desaturated by a FAD2 to form C16:2 or catalysed by an elongase to produce *cis*‐vaccenic acid.

Despite of disputing evidences, palmitoleic acid is gaining attention for its potential to reduce risk factors for coronary heart disease, type 2 diabetes and other obesity‐related diseases (Bernstein *et al*., 2014; Hiraoka‐Yamamoto *et al*., 2004). Further, it has not only high oxidative stability, but also lower melting points than oleic acid, which makes it desirable for use as feedstock of biodegradable lubricants or as a valuable precursor for linear low density polyethylene products (Ohlrogge, 1994; Rybak *et al*., 2008). In sunflower, it has been reported that SAD activity towards stearoyl‐ACP was about 100‐fold higher than for palmitoyl‐ACP (Salas *et al*., 2004). Therefore, it is likely that the low specificity of cotton SAD towards palmitoyl‐ACP could be the bottleneck for raising palmitoleic acid level in HP cotton seed oil. This might be overcome by co‐expressing a modified SAD with enhanced specificity towards palmitoyl‐ACP, together with the down‐regulation of *ghKAS2* expression. In a *KAS2*‐suppressed Arabidopsis, a modified plastidial desaturase and an extraplastidial palmitate Δ9 desaturase yielded a mean accumulation of ~67% palmitoleic acid and *cis*‐vaccenic acid, with individual lines showing greater than 71% in the best engineered line (Nguyen *et al*., 2010). It could be envisaged that as an annual crop amendable to genetic modification, cotton seed oil rich in palmitic acid may become the launching pad for further genetic modification to produce valuable palmitoleic acid *in planta*.

In cotton seed, a relatively smaller proportion of C16 fatty acids are converted to their dienoic form than are the C18 fatty acids. This might be caused by the cotton FAD2 which had a strong substrate preference to oleic acid in relative to palmitoleic acid on PC. A FAD2 that has high substrate specificity for palmitoleic acid has been reported in safflower (Cao *et al*., 2013). However, such a palmitate preference has not yet been identified in cotton *FAD2* that is comprised of only 4 gene members in contrast to a staggering 11 members as reported in safflower (Cao *et al*., 2013). *Cis*‐vaccenic acid is clearly an elongation product of palmitoleic acid, but it remains to be resolved whether the residual KASII in plastids, or the cytoplasmic β‐ketoacyl‐CoA synthase in eukaryotic pathway is the catalysing enzyme (Nguyen *et al*., 2010).

In addition to chain length and degree of unsaturation of fatty acids, it is now recognised that the positional composition of TAG may be an important determinant of metabolic availability. It has been hypothesised that the saturated fatty acids on the *sn*‐2 position are hypercholesterolaemic, but neutral on cholesterol metabolism when on the *sn*‐1 or *sn*‐3 positions (German and Dillard, 2004; Karupaiah and Sundram, 2007). A plausible mechanism to support this hypothesis is that pancreatic lipase and lipoprotein lipase preferentially hydrolyse the fatty acids in the *sn*‐1 and *sn*‐3 position and produce a 2‐MAG that is preferentially transported to liver for LDL cholesterol metabolism (Berry, 2009). Therefore, despite the high level of palmitic acid, the HP cotton seed oil may not be nutritionally undesirable due to its low level of palmitic acid at the *sn*‐2 position in TAG.

We have previously generated HO and HS cotton genotypes using RNAi down‐regulation of *ghFAD2‐1* and *ghSAD‐1,* respectively (Liu *et al*., 2002). The HS cotton seed oil, albeit having a significantly raised melting point compared to the conventional cotton seed oil, contained insufficient saturated fatty acids to provide the required texture and spread ability for direct use as a solid fat unless used in combination with another appropriate fat (unpublished data). Therefore, raising palmitic acid content in the HO and HS genotypes has a clear advantage in providing functionality required for the food industry, especially in the area of solid fat applications, such as margarine, shortening and confectionary industries. In addition, simultaneously raised levels of palmitic, stearic and oleic acids could be potentially useful for mimicking the unique TAG profile of cocoa butter that is in high demand by the ever expanding confectionary, cosmetic and pharmaceutical industries. Cocoa butter is one of the most highly valued plant lipids in commerce because of its melting curve properties conferred by symmetrical TAGs such as *sn*‐1,3 distearoyl *sn*‐2 oleoyl glycerol (SOS), *sn*‐1 palmitoyl *sn*‐2 oleoyl *sn*‐3 stearoyl glycerol (POS) and *sn*‐1,3 dipalmitoyl *sn*‐2 oleoyl glycerol (POP).

We have demonstrated here that both palmitic and oleic acids could be raised simultaneously in cotton seed oil by crossing the HP and HO lines. This is consistent with the HP/HO sunflower mutant CAS‐12 that produces oil with 30% palmitate and 40% oleate (Salas *et al*., 2004). In contrast, the combination of HP and HS trait appeared to be troublesome as the stearic acid level in the hybrid was only 4% compared to about 30% in its HS parent while palmitic acid was retained at a similar level as its HP parent. In this study, we have shown that such a biased accumulation of saturated fatty acids occurred in both PC and TAG lipids. Although the oil biosynthesis pathway is made of discrete elements, it has been established that there are interactions between such components expediting the dynamic channelling of the metabolic flux through fatty acid elongation, desaturation and TAG assembly (Roughan and Ohlrogge, 1996). Several hypotheses could be proposed to explain why the HP cotton is not able to accumulate stearic acid to a high level. These include the low enzyme specificity of downstream enzymes needed for stearic acid incorporation, and FatB's substrate preference towards palmitic acid. The competition between the two saturated fatty acids for their esterification on TAG molecule might be eased by introducing a FatB enzyme or acyltransferases with substrate preference towards stearic acid.

In summary, we report here that expression of RNAi construct targeting the down‐regulation of *KAS2* expression led to substantial increase in palmitic acid and its derivative C16 unsaturated fatty acids at the expense of C18 fatty acids in cotton seed oil. A slight yet significant reduction in oil content has been observed in both HP lines, but seed germination was unaffected. Simultaneous increase of both palmitic acid and oleic acid was also successful. These novel cotton genotypes may provide a temperate vegetable oil, derived from an annual crop, which could potentially replace imported palm oil in many parts of the world. Additional efforts are required for further enhancement of stearic acid in the HP/HO background that may have a clear advantage in providing functionality required for valuable cocoa butter substitute.

## Experimental procedures

### Plant material and growth conditions

Upland cotton (*G. hirsutum*) cv Coker315 was used for gene transformation. Homozygous transgenic cotton lines including HO‐30 and HS‐35 were generated as previously described (Liu *et al*., 2002). Plants were cultivated in greenhouse conditions at 28/18 °C (day/night), with 16‐h photoperiod.

### Cloning cotton *ghKAS2* genes

The cotton *KASII* DNA sequences were isolated from a developing cotton seed cDNA library using a DNA fragment corresponding to the first 400 nucleotide of the coding region of *KASII* gene from Arabidopsis (AF318307) as a probe. Two different *KASII* cDNAs, *ghKASII‐A* and *ghKASII‐B,* were isolated from this cDNA library as previously described (Liu *et al*., 1999).

### Design of RNAi gene silencing construct targeting *ghKAS2*

**Figure 8:**
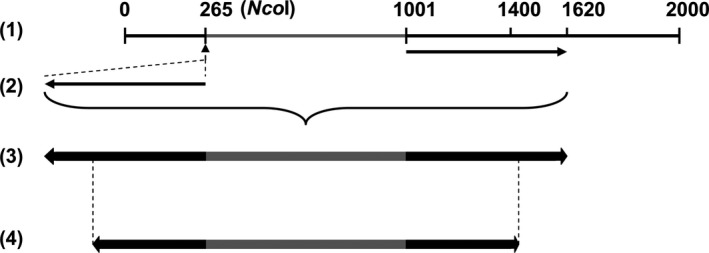
A diagram showing the selection process of trigger DNA sequence for the inverted repeat configuration in RNAi‐*ghKAS2* construct. (1) The DNA sequence corresponding to nucleotides from 1001 to 1620 of *ghKAS2‐1* was PCR amplified and (2) inserted at the *Nco*I site at nt 265 of *ghKAS2‐1*, in an antisense orientation and therefore resulted in the inverted repeat structure as shown in (3). The first 400 bp of the repeated DNA sequences and its adjacent 750‐bp spacer were PCR amplified to create the final RNAi trigger DNA sequence (4).

We designed an RNAi cassette consisting of inverted repeat structure of partial *ghKAS2‐1* DNA sequences driven by a seed‐specific promoter derived from soya bean *lec1* gene (Cho *et al*., 1995). To assemble this RNA cassette, as shown in Fig. 8, we firstly obtained the trigger DNA fragment corresponding to nucleotides from 1001 to 1620 of *ghKAS2‐1* by PCR using a pair of primers incorporating the *Nco*I site (underlined) at both ends: 5′‐CCATGGCTAATCGCGATGGATTTGTCATGG‐3′, and 5′‐CCATGGCTCTTCGGCCAAATGTAAGAAA‐3′. The amplified PCR fragment was then inserted at the *Nco*I site at nt‐265 of *ghKAS2‐1*, in an antisense orientation. A DNA fragment consisting of 400 bp of the inverted repeats in a head to head fashion, together with 750 bp adjacent DNA sequence acting as a spacer, was amplified by PCR using the following single primer that is present at the both sides of the inverted repeats and with an additional *Sma*I restriction site (underlined): 5′‐CCCGGGCGTATTGCCTGTACCGTTGC‐3′. The resulting inverted repeat structure was then inserted at the *Sma*I site between the lectin promoter and terminator sequences (Cho *et al*., 1995) in a binary vector using *NPTII* gene as the selectable marker for gene transformation.

### Transformation of cotton

The above described binary construct was transformed to *Agrobacterium tumefaciens* strain AGL1 and used to transform cotton variety Coker 315 as described by Liu *et al*. (2002). Briefly, cotyledons excised from cotton seedlings were used as explants and were infected and co‐cultivated with *A. tumefaciens* harbouring the RNAi‐*ghKAS2‐1* construct. Healthy calli derived from the cotyledon explants were selected by growing on MS medium containing kanamycin until somatic embryo formation. Plantlets developed from the somatic embryos were subsequently transferred to soil and maintained in a glasshouse once leaves and roots were developed, with 28/20 °C (day/night) growth temperature.

### Real‐time RT‐qPCR

RNAs from developing embryos of homozygous T_4_ KIR‐1, KIR‐10 plants were isolated using QIAEX Plant RNA Miniprep kit (Qiagen, Hilden, Germany). The gene expression patterns were studied with RT‐qPCR carried out in triplicate using Platinum SYBR Green qPCR SuperMix (BioRad, Hercules, CA) and run on ABI 7900HT Sequence Detection System. Each 10‐μL PCR contained 20 ng of total RNA template, 800 mm each of the forward and reverse primers, 0.25 μL of reverse transcriptase, 5 μL One‐step RT‐PCR master mix reagents. PCR was carried out at following conditions with an initial cycle at 48 °C for 30 min, and 95 °C for 10 min, followed by 40 cycles of 95 °C for 15 s and 60 °C for 60 s. The following primers were designed such that amplifying a fragment from the *ghKAS2‐1* gene (5′‐CCAACAAAGACGTGGGCTTGA‐3′ and 5′‐GGAGGCTTCTTCTTCGTTGTAA‐3′) would give a 290‐bp fragment; and amplifying a *ghKAS2‐2* gene (5′‐TCCAAACCCAAGTCCTCAAAACTC‐3′ and 5′‐AGGAGGTTTCTGTTTTGTCATG‐3′) would give rise to a 393‐bp fragment. Cotton unibiquitin‐14 gene (*ghUBQ14,* GenBank accession number: DW505546) was used as an internal reference gene (Zhang *et al*., 2013). The primers for *ghUBQ14* are sense: 5′‐CAACGCTCCATCTTGTCCTT‐3′ and antisense: 5′‐TGATCGTCTTTCCCGTAAGC‐3′. The calculations were made using the comparative CT method as reported (Livak and Schmittgen, 2001). The data are presented as means ± SD of three reactions performed on independent 96‐well plates.

### Oil content analysis, lipid separation and quantification

Seed oil content was quantified by Oxford MQC benchtop nuclear magnetic resonance analyser (NMR) (Oxford Instruments, Oxford, UK). Three‐gram samples of cotton seed were measured and equilibrated to 40 °C for 2 h prior to acquisition of NMR data. The experiment was carried out in triplicate. Two‐way ANOVA was conducted using Microsoft Excel, and LSD test was applied at 5% probability level to compare the differences among treatment means.

Fatty acid profiles of cotton seed oil were determined by GC‐FID analysis of prepared seed oil fatty acid methyl esters (FAME). TAG and polar lipids were fractionated from total lipids on thin layer chromatography (TLC) plates Silica gel 60 (Merck Millipore, Darmstadt, Germany) using hexane/diethyl ether/acetic acid (50/50/1, by volume). Lipid bands were visualised by spraying 0.001% primuline made in 80% acetone in water, and individual lipid classes were identified by running authentic standards on the same TLC plate. Fatty acid profiles of TAG and total polar lipids were determined by incubating corresponding silica bands in 1 N methanolic HCl and by analysing corresponding FAME by GC as above.

Positional distribution of the fatty acids in TAG was analysed by treatment with TAG lipase derived from *Rhizopus arrhizus* (Fluka, Buchs, Switzerland). Purified TAG (1 mg) in chloroform was transferred to a glass vial, evaporated under nitrogen and mixed strongly using a vortex with 2 U *Rhizopus* lipase dissolved in 0.4 mL 0.1 m Tris‐HCl (pH 7.7) and 5 mm CaCl_2_ for 1 min. The reaction was stopped by adding 50 μL 6 m HCl, and lipid was extracted using 1.5 mL CHCl_3_/MeOH (2/1, by volume). The lipid was fractionated into TAG, DAG, sn‐1/3‐MAG, sn‐2‐MAG and FFA using 15 cm 2.3% boric acid TLC (silica gel‐60, Merck Millipore) and hexane/diethyl ether/acetic acid (50/50/1, by volume) solvent mixture. FAMEs of individual lipid classes were prepared directly by collecting the lipid bands in silica gel isolated from the TLC plate into glass vials and by incubation in 1 N methanolic HCl at 80 °C for 2 h prior to GC analysis.

PC was fractionated from the polar lipid pool using TLC plates Silica gel 60 (Merck Millipore) and chloroform/methanol/acetic acid/water (90/15/10/3, by volume) as the solvent system. Lipid classes were visualised and identified as above, and PC was extracted from silica using chloroform/methanol/water (2/1/1, by volume). Positional distribution of fatty acids in PC was determined by the treatment of 0.1 mg of lipid in 3 U of phospholipase A_2_ derived from honey bee (Sigma‐Aldrich, St. Louis, MO) as described by Williams *et al*. (1995). Lipid was further purified using chloroform/methanol/0.1 m KCl (2/1/1, by volume), and it was fractionated into PC, lyso‐PC and free fatty acids by TLC (Silica gel 60, MERCK) in chloroform/methanol/acetic acid/water (68/22/6/4, by volume) as solvent system. FAMEs of individual lipid bands were prepared and analysed as above.

### Primary assessment of seed germination rate

To evaluate seed germination response to temperature, 30 seeds from each of the two HP lines, together with the untransformed control Coker 315, were evenly spaced on moist filter paper in a 200 × 100 mm plastic tray with cover and placed in incubators with cool (18 °C) and warm (28 °C) temperatures. The filter paper was kept moist for the duration of the experiment by adding distilled water when required. Seeds were incubated for 5 days at each temperature and were counted as germinated when the radicle protruded beyond the seed coat and elongated to at least 1 cm in length. The experiment was run in quadruplicate (*n* = 4). Two‐way ANOVA was conducted using Microsoft Excel, and LSD test was applied at 5% probability level to compare the differences among treatment means.

## Supporting information


**Table S1**. Two way ANOVA of oil content in WT, KIR‐1, KIR‐10 across two generations (T_4_ and T_5_).
**Table S2**. Two way ANOVA of germination rate in WT, KIR‐1, KIR‐10 at cool (18 °C) and warm (28 °C) temperatures.
