# Supplementary material for: Genetic enhancement of palmitic acid accumulation in cotton seed oil through RNAi down‐regulation of ghKAS2 encoding β‐ketoacyl‐ACP synthase II (KASII)
Source: Plant Biotechnol J. 2016 Sep 7;15(1):132–43. doi: 10.1111/pbi.12598 (PMC5253470; doi:10.1111/pbi.12598)
Supplement: Supplementary file 1 — Table S1. Two way ANOVA of oil content in WT, KIR‐1, KIR‐10 across two generations (T4 and T5). Table S2. Two way ANOVA of germination rate in WT, KIR‐1, KIR‐10 at cool (18 °C) and warm (28 °C) temperatures. [file PBI-15-132-s001.docx]

Supplemental Tables

Table S1. Two way ANOVA of oil content in WT, KIR-1, KIR-10 across two generations (T_4_ and T_5_)

| *Source of Variation* | *SS* | *df* | *MS* | *F* | *P-value* | *F crit* |
| --- | --- | --- | --- | --- | --- | --- |
| Genotype | 11.55283473 | 2 | 5.776417366 | 15.82304947 | 0.00043193 | 3.885293835 |
| Generation | 1.537525892 | 1 | 1.537525892 | 4.21166732 | 0.06262413 | 4.747225347 |
| Interaction | 0.426470133 | 2 | 0.213235067 | 0.58410409 | 0.57270025 | 3.885293835 |
| Within | 4.380761656 | 12 | 0.365063471 |  |  |  |
|  |  |  |  |  |  |  |
| Total | 17.89759241 | 17 |  |  |  |  |

Table S2. Two way ANOVA of germination rate in WT, KIR-1, KIR-10 at cool (18 °C) and warm (28 °C) temperatures

| *Source of Variation* | *SS* | *df* | | *MS* | *F* | *P-value* | *F crit* |
| --- | --- | --- | --- | --- | --- | --- | --- |
| Genotype | 8.333333 | | 2 | 4.166667 | 0.236842 | 0.791536 | 3.554557 |
| temperature | 1350 | | 1 | 1350 | 76.73684 | 6.57E-08 | 4.413873 |
| Interaction | 19.44444 | | 2 | 9.722222 | 0.552632 | 0.584892 | 3.554557 |
| Within | 316.6667 | | 18 | 17.59259 |  |  |  |
|  |  | |  |  |  |  |  |
| Total | 1694.444 | | 23 |  |  |  |  |
